# Impulsive traits and dual pathology in patients with depression and alcohol dependence, a case report

**DOI:** 10.1192/j.eurpsy.2022.1179

**Published:** 2022-09-01

**Authors:** M. Huete Naval, L. Reyes Molón, C. Regueiro Martín-Albo, R. Galerón, E. Herrero Pellón, P. Albarracin

**Affiliations:** 1 Hospital Clínico San Carlos, Institute Of Psychiatry And Mental Health, Madrid, Spain; 2 Hospital Clínico San Carlos, Instituto De Psiquiatría Y Salud Mental, Madrid, Spain; 3 Hospital Universitario Clínico San Carlos, Psychiatry And Mental Health, Madrid, Spain

**Keywords:** Depression, Impulsivity, Alcohol dependence, dual pathology

## Abstract

**Introduction:**

Alcohol dependence is one of the most frequent comorbidities in depression. Multiple environmental and neurobiological factors are directly involved in these diseases. In particular, impulsivity is present in many patients with dual pathology and may play a relevant role in its causes, clinical manifestations and prognosis.

**Objectives:**

To review the relationship between impulsive traits and dual pathology in patients with depression and alcohol dependence.

**Methods:**

Presentation of a clinical case supported by a non- systematic review of literature containing the key-words “impulsivity”, “depression” and “alcohol dependence”.

**Results:**

This is a case report of a 43-year-old male with a known history of alcohol dependence and recurrent depression. Interestingly, the patient has a family history of bipolar disorder and alcohol abuse disorder on the paternal side, and frontotemporal dementia on the maternal side. He currently presents a depressive episode associates associated with a significant increase in alcohol consumption. The patient has presented prominent impulsive traits since adolescence that have been aggravated in recent years. This lack of impulse control is described as one of the most relevant factors in relapses in alcohol consumption. Multiple studies correlate the lack of impulse control with a worse prognosis in both alcohol dependence (greater probability of relapses and resistance to treatment) and depression (increased suicide risk). Likewise, an increase in cognitive impulsivity has been observed during depressive episodes, characterized by an inability to inhibit behaviors that have already begun and poor planning capacity, which could lead to a worsening of alcohol abuse.

**Conclusions:**

Impulsivity traits are related to a worse prognosis in dual pathology due to alcohol and depression, and may present common etiopathogenic mechanisms.

**Disclosure:**

No significant relationships.

